# Unexpected species diversity in electric eels with a description of the strongest living bioelectricity generator

**DOI:** 10.1038/s41467-019-11690-z

**Published:** 2019-09-10

**Authors:** C. David de Santana, William G. R. Crampton, Casey B. Dillman, Renata G. Frederico, Mark H. Sabaj, Raphaël Covain, Jonathan Ready, Jansen Zuanon, Renildo R. de Oliveira, Raimundo N. Mendes-Júnior, Douglas A. Bastos, Tulio F. Teixeira, Jan Mol, Willian Ohara, Natália Castro e Castro, Luiz A. Peixoto, Cleusa Nagamachi, Leandro Sousa, Luciano F. A. Montag, Frank Ribeiro, Joseph C. Waddell, Nivaldo M. Piorsky, Richard P. Vari, Wolmar B. Wosiacki

**Affiliations:** 1 Division of Fishes, Department of Vertebrate Zoology, MCR 159, National Museum of Natural History, PO Box 37012, Smithsonian Institution, Washington, DC, WA 20013-7012 USA; 2 Department of Biology, University of Central Florida, Orlando, FL 32816 USA; 3 Cornell University Museum of Vertebrates, Department of Ecology and Evolutionary Biology, Cornell University, Ithaca, NY 14850 USA; 4 Laboratório de Ecologia e Conservação, Universidade Federal do Pará, Instituto de Ciências Biológicas, Belém, Pará Brazil; 5 Department of Ichthyology, The Academy of Natural Sciences of Drexel University, 1900 Benjamin Franklin Parkway, Philadelphia, PA 19103 USA; 6 Muséum d’histoire naturelle, Département d’herpétologie et d’ichtyologie, route de Malagnou 1, case postale 6434, CH-1211 Genève 6, Switzerland; 7 Laboratório de Lepidopterologia e Ictiologia Integrada, Centro de Estudos Avançados da Biodiversidade, Instituto de Ciências Biológicas, Universidade Federal do Pará, Belém, Pará Brazil; 8 Coordenação de Biodiversidade, Instituto Nacional de Pesquisas da Amazônia, Manaus, Amazonas Brazil; 9 RESEX do Rio Cajari, Instituto Chico Mendes da Conservação da Biodiversidade, Macapá, Amapá, Brazil; 10 Museu de Zoologia da Universidade de São Paulo, Laboratório de Ictiologia, São Paulo, São Paulo Brazil; 11 Anton de Kom University of Suriname, Paramaribo, Suriname; 12 Museu Paraense Emílio Goeldi, Caixa Postal 399, 66040-170 Belém, Pará Brazil; 13 Laboratório de Citogenética, Centro de Estudos Avançados da Biodiversidade, Instituto de Ciências Biológicas, Universidade Federal do Pará, Belém Pará, Brazil; 14 Laboratório de Ictiologia, Faculdade de Ciências Biológicas, Universidade Federal do Pará, Altamira Pará, Brazil; 15 Instituto de Ciências e Tecnologia das Águas, Universidade Federal do Oeste do Pará, Campus Amazônia, Santarém Pará, Brazil; 16 Universidade Federal do Maranhão, Departamento de Biologia, Laboratório de Ecologia e Sistemática de Peixes, São Luis, Maranhão Brazil; 17 Present Address: Laboratório de ecologia de peixes, Universidade Federal de Minas Gerias, Institudo de Ciências Biológicas, Belo Horizonte, Minas Gerais Brazil; 18 Present Address: Programa de Pós-Graduação em Biologia de Vertebrados, Pontifícia Universidade Católica de Minas Gerais, Belo Horizonte, Minas Gerais Brazil; 19 Present Address: Laboratório de Ciências Ambientais, Universidade Federal de Rondônia, Presidente Médice, Rondônia, Brazil

**Keywords:** Biodiversity, Taxonomy, Ichthyology

## Abstract

Is there only one electric eel species? For two and a half centuries since its description by Linnaeus, *Electrophorus electricus* has captivated humankind by its capacity to generate strong electric discharges. Despite the importance of *Electrophorus* in multiple fields of science, the possibility of additional species-level diversity in the genus, which could also reveal a hidden variety of substances and bioelectrogenic functions, has hitherto not been explored. Here, based on overwhelming patterns of genetic, morphological, and ecological data, we reject the hypothesis of a single species broadly distributed throughout Greater Amazonia. Our analyses readily identify three major lineages that diverged during the Miocene and Pliocene—two of which warrant recognition as new species. For one of the new species, we recorded a discharge of 860 V, well above 650 V previously cited for *Electrophorus*, making it the strongest living bioelectricity generator.

## Abstract (web summary)

A single species of electric eel, *Electrophorus electricus*, has been described. Here, de Santana et al. show that there are three major lineages of *Electrophorus* distributed across Greater Amazonia and describe two new species, one with a much stronger electric discharge than was previously known.

## Introduction

Is there only one electric eel species? Since Linnaeus’s description of *Electrophorus electricus* 250 years ago^1^, electric eels have fascinated scientists and layperson alike by their capacity to generate strong (~650 V) electric organ discharges (EODs)^2,3^. Strong EODs facilitate hunting, prey capture, and defense, while weaker (~10 V) EODs allow electrolocation and communication^4^. Electric eels inspired the design of Volta’s first electric battery to provide constant current, provide a source of acetylcholinesterase for treating neurodegenerative diseases^5^, and recently encouraged the development of synthetic protocells with natural nanoconductors and capacitators^6,7^, and a stacked hydrogel battery that could be used to power medical implants^8^. Electric eels are also an emerging model for genomic studies of animal electrogenesis^9^. Due in part to their large size [up to 2.5 m^10^], and specialized electrogenic morphology, electric eels have long been assumed to comprise a single species broadly distributed through Greater Amazonia—the superbasin comprising the Amazon, Orinoco, and coastal drainages of the Guianas e.g., refs. ^11,12^.

To test the hypothesis of a single species of *Electrophorus*, we examine 107 specimens from across Greater Amazonia—including the type locality of *E*. *electricus* in Suriname^13^ (Supplementary Data 1). To explore species-level divergences, we adopt the General Lineage Concept (GLC)^14^, which recognizes species as separately evolving metapopulation lineages. The GLC unifies several pre-existing species concepts, which vary in their criteria for identifying the point of lineage divergence during speciation^14^. Practical applications of the GLC seek multiple, congruent lines of evidence for delimiting species, and to this end we subject a large dataset (comprising mitochondrial and nuclear DNA, morphology, and geographical and ecological distributions) to a range of empirical and model-based procedures. Our analyses lead us to conclude that there are three common species of *Electrophorus*, which occupy predominantly allopatric ranges (i.e., occupy different regions) in the Guiana Shield (*E*. *electricus*), Brazilian Shield (*E*. *voltai* sp. nov.) and in the lowland Amazon basin (*E*. *varii* sp. nov.). Here we describe these three species, and discuss their morphology, evolutionary history, and ecology.

## Results and discussion

### Genetic analysis

**Fig. 1:**
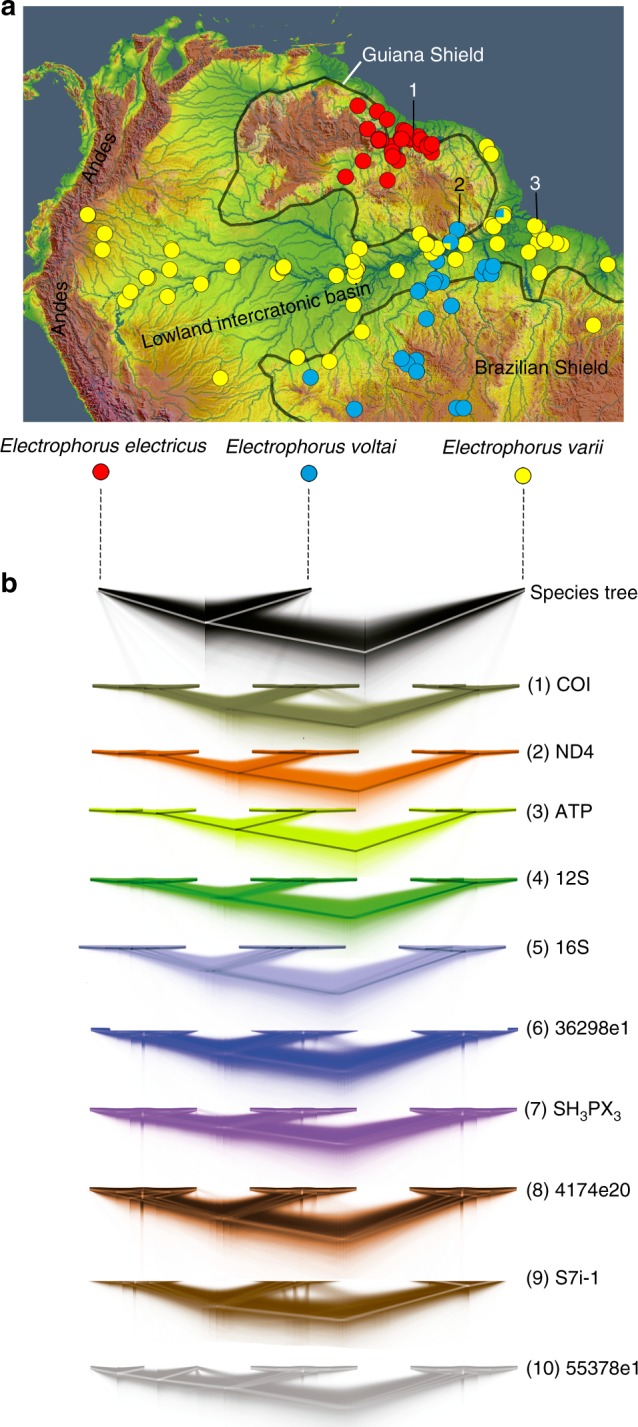
Sampling localities and gene trees for the three species of *Electrophorus*. **a** Map of northern South America showing distributions of sampled records and type localities (indicated by numbers) for three electric eel species: *Electrophorus electricus* (red dots, 1 = Suriname River, Suriname); *E*. *voltai* (blue dots, 2 = Rio Ipitinga, Brazil); and *E*. *varii* (yellow dots, 3 = Rio Goiapi, Brazil). Bicolor dots (blue/yellow) indicate sympatric co-occurrence of *E*. *voltai* and *E*. *varii*. The map was created in ArcGIS (https://www.arcgis.com) with images available at Shuttle Radar Topography Mission, Global Multi-resolution Terrain Elevation Data, and HydroSHEDS database. **b** *BEAST2.4 species tree (top cladogram; 94 specimens: 15 *E*. *electricus*, 41 *E*. *voltai*, 38 *E*. *varii*) based on 5 mitochondrial (trees 1–5; 107 specimens: 19 *E*. *electricus*, 43 *E*. *voltai*, 45 *E*. *varii*) and 5 nuclear genes (6–10; 94 specimens). Higher shading densities represent areas where the majority of trees agree in topology and branch lengths (posterior probabilities >0.99), while lower densities represent areas of uncertainty (Supplementary Data 1)

Phylogenetic analyses based on the mitochondrial *COI* gene resolved three divergent and highly supported lineages corresponding to *E*. *electricus*, and the two proposed new species *E*. *voltai*, and *E*. *varii*—both with Bayesian Inference [posterior probability (PP) >0.95], and Maximum-Likelihood (ML) analysis (bootstrap >0.95; Fig. 1). Estimated evolutionary divergences of *COI*, using Kimura 2-parameter distances, are: 6.6% between *E*. *electricus* and *E*. *voltai*; 9.8% between *E*. *electricus* and *E*. *varii*; and 9.3% between *E*. *voltai* and *E*. *varii*. Intra-specific divergences range from 0.02% in *E*. *electricus* to 0.31 and 0.32% in *E*. *voltai*, and *E*. *varii*, respectively. Interspecific *COI* divergences are also well above the accepted threshold (~2%) used to recognize animal species, including fishes^15^. Finally, sequences were analyzed by pairwise distances to assess intra- and interspecific variation, without a priori species hypotheses, using Automatic Barcoding Gap Discovery (ABGD)^16^. ABGD clustered the sequences into the same three lineages.

Concatenated mitochondrial DNA (*COI*, *ND4*, *ATPase6/8*, *12S* rDNA, and *16S* rDNA) was analyzed with three General Mixed Yule Coalescent (GMYC) models: the Bayesian Poisson tree process (bPTP), single- (SML) and multi-threshold (MML) maximum-likelihood methods, and the Genealogical Sorting Index (GSI). The results for GMYC (bPTP, *E*. *electricus* 0.999; *E*. *varii* 0.826; *E*. *voltai* 0.973); SML (3 clusters, *p* = 5.7e-14), MML (3 clusters, *p* = 5.6e-14), and GSI (*gsi* = 1, *p* < 0.001 for all three species) strongly support the same three lineages recovered from *COI*.

Concatenated nuclear DNA (*S7i-1*, *SH*_*3*_*PX*_*3*_, *36298E1*, *4174E20*, and *55378E1*) was analyzed under coalescent-based methods for species delimitation in the Bayesian Phylogenetics and Phylogeography program BP&P v3.2^17^, and by Bayesian posterior probabilities (PP). Results derived from these analyses (Fig. 1) strongly support the same three lineages (PP = 1.0) recovered from *COI* and concatenated mitochondrial DNA.

The full set of ten nuclear and mtDNA markers was analyzed using species delimitation in BP&P and Bayesian Evolutionary Analysis by Sampling Trees [*BEAST2.4]^18^ (Fig. 1). The same three lineages recovered by previous analyses were, again, overwhelmingly supported (PP = 1.0).

### Morphological analysis

**Fig. 2:**
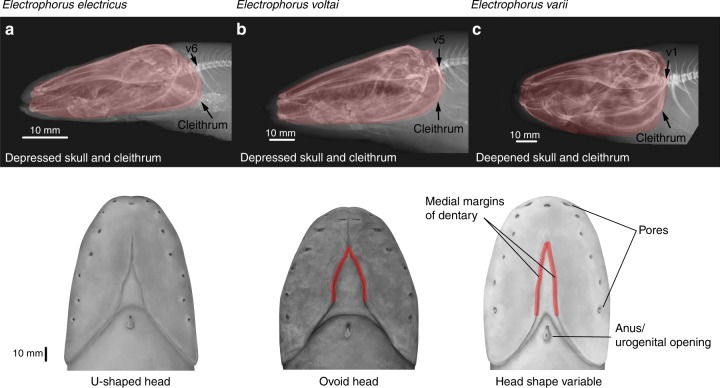
Key morphological features to recognize the three species of *Electrophorus*. Top, radiographs of lateral view of the anterior portion of body (skull and pectoral girdle highlighted red). The cleithrum lies between the fifth and sixth vertebrae (v) in *Electrophorus electricus* (**a**) and *E*. *voltai* (**b**) *versus* first and second vertebrae in *E*. *varii* (**c**). Bottom, illustrations of ventral view of the head, showing key features listed in Diagnoses. **a** top: National Museum of Natural History, NMNH 403765, 300 mm TL, Cuyuni River, Guyana; bottom: NMNH 225576, 1000 mm TL, Corantijn River, Suriname. **b** top: Instituto Nacional de Pesquisas de Amazônia, INPA 39009, 450 mm TL, Teles Pires River, Brazil; bottom: Academy of Natural Sciences of Drexel University, ANSP 197583 (t3539), 1280 mm TL, Xingu River, Brazil. **c** top: NMNH 306677, 450 mm TL, Lago Janauari, Amazon River, Brazil; bottom: NMNH 196634, 1220 mm TL, Amazon River, Brazil

Due to uniform body shape and coloration, neither morphometric analyses of 19 linear body measurements (Supplementary Data 2) nor pigmentation characters unambiguously distinguish the three species of *Electrophorus*. However, a species-level assessment based on characters from the lower jaw, neurocranium, and cleithrum separate specimens of *Electrophorus* into two groups (Fig. 2): those possessing a dorsoventrally depressed skull (*E*. *electricus* and *E*. *voltai*), and those with a deepened skull (*E*. *varii*). The cleithrum lies between vertebrae 5 and 6 in *E*. *electricus* and *E*. *voltai* and between 1 and 2 in *E*. *varii*. We found additional diagnostic differences in head shape (Fig. 2), and non-overlapping ranges in the number of pectoral-fin rays (e.g., 32–38 in *E*. *electricus versus* 20–28 in *E*. *varii*) and lateral-line pores (e.g., 88–101 in *E*. *electricus* versus 124–186 in *E*. *varii* and 112–146 in *E*. *voltai;* for more details, see Diagnoses). These historically overlooked characters unambiguously assign all individuals of *Electrophorus* to the same three species delimited by our genetic analyses.

### *Electrophorus* interrelationships

The interrelationships among *Electrophorus* and outgroup genera are beyond the scope of this paper; however, some of our findings, based on a limited number of outgroup taxa, deserve comment. Our analyses recovered *Gymnotus* as part of an unresolved polytomy, with the genera *Hypopomus* and *Sternopygus* both constituting sister taxa to the polytypic *Electrophorus* (both in ML and in the trimmed terminals (*n* = 3) Bayesian analysis). In the full 113 terminal dataset *Hypopomus* is recovered as sister to *Electrophorus* (see Supplementary Fig. 1). In each of our analyses very long branches subtend all clades. The sampling schema undertaken herein, wherein many terminals within the genus *Electrophorus* are analyzed alongside the proposed sister lineage, i.e., *Gymnotus*^19^, as well as a single species each of *Hypopomus* and *Sternopygus* the resultant topologies are not to be taken as proposal of new inter-generic relationships. Instead the sampling was chosen a priori based on previous work^19^, to calibrate divergence estimates, and provide a diversity of outgroup taxa with which to evaluate the focal taxon *Electrophorus*. Based on previous research it has been proposed that *Electrophorus* is either a member of a monotypic Electrophoridae^11^ or part of the Gymnotidae^19^. Regardless of familial placement and interrelationships the single branch that leads to *Electrophorus*, heretofore a single widespread species (and now comprising the nominal species and two new species) is representative of a unique lineage that is unlike other gymnotiforms.

### Temporal diversification

**Fig. 3:**
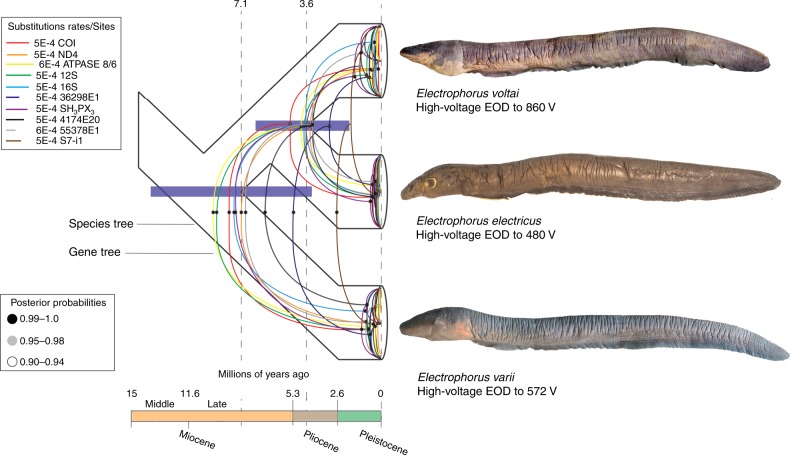
*Electrophorus* tree of life and time of species diversification. Time-calibrated genealogy of *Electrophorus* based on a maximum clade credibility (MCC) species tree derived from *BEAST2.4 analyses of 10 genes (colored lines) and 94 specimens of *Electrophorus* (relaxed molecular clock and uncorrelated lognormal model implemented). Purple bars represent 95% highest posterior density distributions for the estimated divergence time of each major node. Voltage measurements made by us are reported below *E*. *electricus* (National Museum of Natural History, NMNH 225670, 520 mm TL, Corantijn River, Suriname), *E*. *voltai* (Museu Paraense Emílio Goeldi, MPEG 15529; holotype, 1290 mm TL), and *E*. *varii* (MPEG 25422; holotype, 1000 mm TL)

We used estimates for the origin of the Isthmus of Panama^20^ with outgroup taxon sampling in additional gymnotids, i.e., *Gymnotus carapo* (South America) and *G*. *cylindricus* (Central America) (see ref. ^21^) as a calibration point for the multilocus species tree generated by *BEAST2.4, based on Maximum Clade Credibility, with a relaxed clock assumption for the mtDNA loci^22^ and a strict clock for the nDNA^23^. A normal distribution was set to 10.5 Ma (see Time divergence estimates in Methods) and standard deviation of ±1.5 for the outgroup taxa spanning the Isthmus^20^. The resulting time-calibrated genealogies (Fig. 3) estimate the divergence between *E*. *varii* and *E*. *electricus* + *E*. *voltai* to have occurred by the late Miocene (7.1 Ma; 95% highest posterior density: HPD 8.9–5.2 Ma), with subsequent divergence between *E*. *electricus* and *E*. *voltai* in the Pliocene (3.6 Ma; 95% HPD 4.7–2.5 Ma).

### Ecological distributions, biogeography, and divergence events

*Electrophorus electricus* is restricted to the Guiana Shield, and *E*. *voltai* occurs in generally north-flowing rivers of the Brazilian shield and south-flowing rivers of the Guyana shield. In contrast, *E*. *varii* occurs in lowland floodplain and terra-firme systems of the intercratonic Amazon Basin (Fig. 1; Supplementary Data 1). *Electrophorus varii* and *E*. *voltai* co-occur in some streams in the Guiana Shield (Fig. 1). The Miocene divergence of *E*. *electricus* + *E*. *voltai* and *E*. *varii* may reflect ecological specialization to shield versus lowland habitats. Shield streams and rivers are: (1) permanently normoxic (>3 mg/l dissolved oxygen); (2) uniformly low in conductivity (<30 µScm^−1^); and, (3) include rocky substrates, rapids, and waterfalls^12^. In contrast, waters of the lowland Amazon: (1) include low conductivity blackwaters (<30 µScm^−1^) and high-conductivity whitewaters (60–350 µScm^−1^); (2) include permanently normoxic terra-firme streams (>3 mg/l), and seasonally hypoxic floodplains (<0.5 mg/l); and, (3) are slow flowing—with non-rocky substrates and without rapids or falls^12^. Some morphological specializations may have attended divergence into shield versus lowland systems. For instance, the depressed skull of *E*. *electricus* and *E*. *voltai* may represent an adaptation for foraging in rocky substrates or withstanding higher flow—mirroring specializations in other rheophilic (fast-flow-adapted) fishes^24^.

We hypothesize that the divergence of *E*. *voltai* (Brazilian Shield) and *E*. *electricus* (Guiana Shield)—both restricted to low conductivity systems (Fig. 1a)—may have arisen from dispersal barriers imposed by the emergence of the Amazon’s modern (high-conductivity) river-floodplain course in eastern Amazonia (ref. ^25^ describes similar disjunct distributions in other taxa). The separation of the Guiana and Brazilian Shields by a major river-floodplain resulted from the reversal of a paleo west-flowing Amazon to the contemporary east-flowing Amazon during the Miocene-Pliocene. The Amazon River was initiated as a transcontinental river 9.4–9 Ma (late Miocene) by recent estimates^26^, began entrenchment about 6.8 Ma and developed its modern shape from about 2.4 Ma onwards^27^. Notwithstanding debate over the timing of these events^26,28^, our estimated 3.6 Ma (95% HPD 4.7–2.5 Ma) divergence of *E*. *voltai* and *E*. *electricus* (Fig. 3) is coincident with the later stages of the origins of the Amazon’s eastern course.

**Fig. 4:**
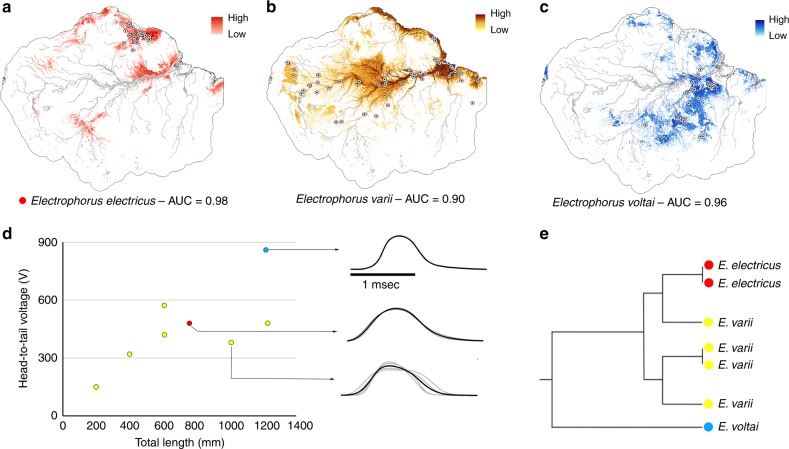
Ecological Niche Model and electric organ discharges for species of *Electrophorus*. Species niche models generated by MaxEnt for Greater Amazonia: **a** *Electrophorus electricus* (red); **b** *E*. *varii* (yellow); and **c** *E*. *voltai* (blue). **d** Measurements of voltage of high-voltage EODs, low-voltage EODs waveforms from Sach’s organ, and posterior one-third of Hunter's organ (grey lines = individually recorded fish, black lines = averaged waveform for each species). **e** Nearest-neighbor hierarchical clustering of prominent time-frequency features of the low-voltage Sach’s organ EOD from seven individuals of *Electrophorus*

Do the geographical and ecological distributions of *Electrophorus* reflect predictive models of niche occupation? We used Ecological Niche Models (ENMs) based on climatic and geomorphological variables to test the premise of divergent habitat requirements for each species of *Electrophorus*. ENMs, based on MaxEnt presence-only algorithms, predicted the potential niche distributions of *Electrophorus* with strong confidence (Area Under the Curve, AUC [≥0.90], Fig. 4a–c). Likewise, observed geographic ranges are significantly influenced by the abiotic environmental factors included in our analyses: seasonality of air temperature (ST) and annual mean temperature (AMT)—strong predictors of flood pulse; altitude (AL), annual mean precipitation (AMP), and flow accumulation (FLA)—strong predictors of aquatic habitat structure, and; soil types (SOT 0, 3, 6, 11)—predictors of water chemistry. The predicted niche area for *E*. *electricus* (Fig. 4a; AUC = 0.98) designates AL (44.7%) and AMT (25.3%) as the strongest contributors to the models. For *E*. *voltai* (Fig. 4c; AUC = 0.96), SOT (35.4%) and FLA (25.4%) contributed most. For *E*. *varii* (Fig. 4b; AUC = 0.90), AL (72.3%) and FLA (13.1%) contributed most. Despite strong performance, ENMs nonetheless generated some over-predictions of ranges (Fig. 4a–c). For instance, they inaccurately predicted *E*. *electricus* to occur in portions of the lowland Amazon basin between the Guiana and Brazilian Shields (Fig. 4a). Likewise, ENMs incorrectly predicted the occurrence of *E*. *varii* in the northern portion of the Guiana Shield (Fig. 4b).

A hypothesis of niche divergence among *Electrophorus* species was corroborated by multivariate analyses of variance (MANOVA) of climatic and geomorphological data. MANOVA confirmed significant differences among the niches modeled for each species (Pillai’s lambda = 1.1092, F = 10.414, *P* < 0.001).

In summary, while best regarded as approximations, our ENMs for *Electrophorus* support a hypothesis of divergent niche requirements and geographical ranges corresponding to distinct ecological conditions.

All the three species of *Electrophorus* have a low-voltage (Sachs’ organ/posterior Hunter’s organ) *electric organ discharges* (EODs) and high-voltage (main/anterior Hunter’s organ) with a head-positive monophasic waveform. The low-voltage EOD varies in duration across the species as follows (Fig. 4d): *E*. *electricus* (2.03–2.19 ms, n = 2), *E*. *varii* (1.24–1.78 ms, n = 4), and *E*. *voltai* (1.72 ms, n = 1). The high-voltage EOD (Fig. 4d) ranges from 480 V at 760 mm TL, n = 1, in *E*. *electricus*; 151 V (200 mm TL) to 572 V (609 mm TL), n = 4, in *E*. *varii*; and 860 V at 1219 mm TL, n = 1, in *E*. *voltai*.

To explore similarity in EOD waveform structure between the three species of *Electrophorus* (Fig. 4d) we extracted prominent time-frequency features from all available low-voltage Sach’s organ EOD waveform recordings using the discrete wavelet transform (DWT) and subjected the resulting matrix of DWT coefficients to dimension reduction by pairwise ANOVA; see refs. ^29,30^. Finally, we subjected this reduced matrix to a nearest-neighbor (single linkage) multivariate hierarchical clustering procedure.

Nearest-neighbor clustering analysis (Fig. 4e) demonstrated that the Sach’s organ EOD waveform structures of *E*. *electricus* and *E*. *varii* cluster together, while the (single recorded) EOD of *E*. *voltai* is dissimilar to those of *E*. *electricus* + *E*. *varii*—primarily due to its shorter duration. The results of this clustering analysis were also congruent with measurements of the multivariate Mahalanobis distance (D^2^) between the centroids of each species: D^2^ for *E*. *electricus* to *E*. *varii* = 25, D^2^ for *E*. *electricus* to *E*. *voltai* = 375; D^2^ for *E*. *varii* to *E*. *voltai* = 540. The hierarchical classification of EOD waveform structure in Fig. 4e is not congruent with the phylogeny of *Electrophorus* (Fig. 3), suggesting that distances in EOD signal-space are not correlated to phylogenetic distance as would be expected if EOD structure evolves via non-adaptive drift. Instead, because the low-voltage Sach’s organ EOD may facilitate species-recognition (as documented in weakly-electric gymnotiforms^31^, and because *E*. *voltai* and *E*. *varii* co-occur in geographical sympatry in parts of the lower Amazon, we hypothesize that the EODs of *E*. *voltai* may have diverged from that of *E*. *varii* as an adaptive response to costs associated with heterospecific mismating events (i.e., reproductive character displacement [RCD]; see ref. ^32^. Nonetheless, we stress that these analyses are based on small sample sizes (*E*. *electricus*, *n* = 2; *E*. *varii*, *n* = 4; *E*. *voltai*, *n* = 1). A thorough test of the RCD hypothesis will require a much larger dataset of signals with an expanded geographical coverage.

### Systematic biology

*Electrophorus* Gill, 1864

*Electrophorus* ref. ^33^: 152. Type species: *Gymnotus electricus* Linnaeus, 1766. Type by monotypy. Gender: masculine.

*Electrophorus electricus* (Linnaeus, 1766)

*Gymnotus electricus*^1^; *Gymnoti tremuli* ref. ^34^: 27, pl. 3; *Gymnotus tremulus* ref. ^35^: 111; *Gymnotus electricus* ref. ^1^: 427; *Gymnotus regius* ref. ^36^: 273.

**Diagnosis:** Ten nucleotides in COI (BOL-COIfishF1/R1; 569-bp fragment): G(8), A(50), T(76), T(77), T(107), C(119), C(182), G(272), G(494), A(560). Ventral outline of head U-shaped, widest at terminus of branchial opening (Fig. 2a) and lateral-line pores 88–101 (versus ovoid, widest anterior to branchial opening, Fig. 2b; 112–146 in *E*. *voltai*). Distinguished by skull depressed, cleithrum lies between vertebrae 5 and 6 (Fig. 2a), pectoral-fin rays 32–38, and lateral-line pores 88–101 (versus skull deep, cleithrum lies between vertebrae 1 and 2, Fig. 2c, 20–28, and 124–186 in *E*. *varii*, respectively).

**Fig. 5:**
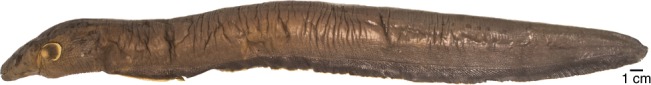
Lateral view of *Electrophorus electricus*. National Museum of Natural History, NMNH 225670, 520 mm TL. Corantijn River, Suriname

**Description:** Species illustrated in Figs. 2, 3, and 5. Maximum size examined specimens 1000 mm TL (total length). Morphometric and meristic data in Supplementary Data 2. Body elongate; sub-cylindrical at pectoral girdle, progressively compressed posteriorly. Mouth superior. Scales absent. Anal and caudal fins seamlessly conjoined. Anus and urogenital papilla separated, located anterior to ventral margin of branchial opening. Head and body color brown to blackish. Clear band along body, below lateral line, variably present.

Low-voltage (Sachs’ organ) EOD and high-voltage (main/Hunter’s organ) with head-positive monophasic waveform. Low-voltage EOD 2.03–2.19 ms duration, *n* = 2 (Fig. 4d). High-voltage EOD 480 V at 760 mm TL, *n* = 1 (Fig. 4d). For distribution see Fig. 1a and Supplementary Data 1.

*Electrophorus varii*, sp. nov. de Santana, Wosiacki, Crampton, Sabaj, Dillman, Mendes-Júnior and Castro e Castro

**Holotype:** MPEG 25422, 1000 mm TL; Goiapi River, Marajó Island, Pará, Brazil.

**Paratypes:** INPA 46378, INPA 46379 (3); MHNG 2748.083; MPEG 30480 (3).

**Etymology:** In honor of Richard Peter Vari (1949–2016) for his contributions to ichthyology.

**Diagnosis:** Eleven nucleotides in COI: A(64), A(80), G(146), G(164), T(190), A(251), A(467), C(512), T(517), A(536), and C(569). Pectoral-fin rays 20–28 and lateral-line pores 124–186 (versus 32–38, and 88–101, respectively, in *E*. *electricus*). Distinguished by head narrow, Fig. 2a (versus wide, Fig. 2b, in *E*. *voltai*; distance between medial margins of contralateral dentaries at transverse through last two ventral pores 2–3 times shorter in *E*. *varii* than *E*. *voltai*, Fig. 2a, b), skull deep, cleithrum lies between vertebrae 1 and 2, Fig. 2b (versus skull depressed, cleithrum lies between vertebrae 5 and 6, in both *E*. *electricus* and *E*. *voltai* Fig. 2a, c).

**Fig. 6:**
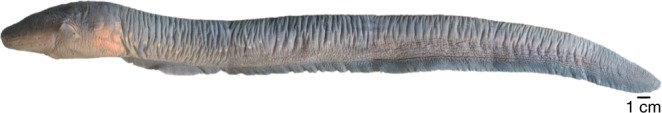
Lateral view of *Electrophorus varii* sp. nov. Holotype, Museu Paraense Emílio Goeldi MPEG 25422, 1000 mm TL. Goiapi River, Brazil

**Description:** As for *E*. *electricus*, except as noted in Diagnosis and except clear band along body always absent. Species illustrated in Figs. 2, 3, and 6. Maximum size examined specimens 1485 mm TL. Morphometric and meristic data in Supplementary Data 2. Low-voltage EOD duration 1.24–1.78 ms (Fig. 4d), high-voltage EOD 151 V (200 mm TL) to 572 V (609 mm TL) (Fig. 4d), *n* = 4. For distribution see Fig. 1a; Supplementary Data 1.

*Electrophorus voltai*, sp. nov. de Santana, Wosiacki, Crampton, Sabaj, Dillman, Castro e Castro, Bastos and Vari

**Holotype:** MPEG 15529, 1290 mm TL; Ipitinga River, Almerim, Pará, Brazil.

**Paratypes:** ANSP 197583 (4), INPA 50453 (8); MPEG 24793, MPEG 30365-71; MZUSP 116410 (2); MZUSP 116421 (5).

**Etymology:** In honor of Alessandro Giuseppe Antonio Anastasio Volta (1745–1827).

**Diagnosis:** Eight nucleotides in COI: A(25), C(29), C(50), C(86), C(140), A(230), A(338), and C(545). Ventral outline of head ovoid, widest anterior to branchial opening (Fig. 2b) and lateral-line pores 112–146 (versus U-shaped, Fig. 2a, 88–101 in *E*. *electricus*). Distinguished by skull depressed, cleithrum lies between vertebrae 5 and 6; and head wide (versus skull deep, cleithrum lies between vertebrae 1 and 2, Fig. 2b, and head narrow in *E*. *varii*), and distance between medial margins of contralateral dentaries at transverse through last two ventral pores 2–3 times longer in *E*. *voltai* than in *E*. *varii*, Fig. 2b, c.

**Fig. 7:**
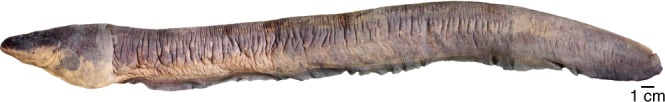
Lateral view of *Electrophorus voltai* sp. nov. Holotype, Museu Paraense Emílio Goeldi MPEG 15529, 1290 mm TL. Ipitinga River, Brazil

**Description:** As for *E*. *electricus*, except as noted in Diagnosis. Species illustrated in Figs. 2, 3, and 7.

Maximum size examined specimens 1711 mm TL. Morphometric and meristic data in Supplementary Data 2. Low-voltage EOD duration 1.72 ms, (Fig. 4d), high-voltage EOD 860 V at 1219 mm TL (Fig. 4d), *n* = 1. For distribution see Fig. 1a; Supplementary Data 1.

### Prospectus

We document hidden species-level diversity in the electric eel illustrating how widespread conspicuous species (over 2 m in total length) can go overlooked even in a long-known model organism from one of Earth’s biodiversity hotspots. Our results demonstrate the invaluable use of multi-disciplinary approaches to explore and understand biodiversity. We further expand our knowledge on the incredible strength of high-voltage electric organ discharges (EOD’s) produced by living organisms; herein demonstrated at 860 V. The discharge recorded of *E*. *voltai* is distinctly higher than any voltage previously cited for *Electrophorus*^2,3^, making it the strongest bioelectricity generator known. We also describe additional species for investigating models in bioelectrogenesis. Future field-based investigations of EOD diversity coupled with studies of the physiological and cellular basis of electrogenesis become an additional priority and may reveal evidence for reproductive isolation and speciation based on variation in communication signals^32^, as well as shed light on the role of *Electrophorus* as electroreceptive predators^37^. Recently, genomic and proteonomic tools have been used to greatly enhance our knowledge of the convergent origins of strong electric discharges^9^. The results shown here suggest that sequencing and comparing the genomes of these three electric eel species will yield further advances towards the origins of, and underlying structures responsible for generation and output of high-voltage electric discharges^38^. Assessment of further population and/or species diversity in *Electrophorus* will follow this study, based on the incorporation of additional specimens from targeted areas (including the upper Negro and Orinoco drainages). A comprehensive understanding of *Electrophorus* could also reveal a hidden variety of enzymatic or bioelectrogenic functions of interest to the broader scientific community^39^.

## Methods

### Taxon sampling and specimen collection

To test the hypothesis of a single species of *Electrophorus*, we examined 107 specimens (all sequenced for mitochondrial DNA, mtDNA, and 94 specimens for nuclear DNA, nDNA) from across Greater Amazonia including the type locality of *E*. *electricus* in Suriname (Supplementary Data 1). Outgroup species were *Gymnotus carapo*, *G*. *choco*, *G*. *cylindricus*, *G*. *pantherinus*, *Hypopomus artedi*, and *Sternopygus macrurus* (Supplementary Data 3). Specimens were collected and sampled in the field according to the Animal Care and Use standards of the depository institutions and the countries of origin of the tissue samples used in the DNA analyses. In addition, tissues and/or specimens were received from multiple institutions in North and South America and Europe following pertinent Material Transfer Agreements and the national and international protocols for the shipment of materials. Specimens were euthanized and muscle or fins removed and stored in 95% ethanol. All voucher specimens are deposited in the institutions listed in the abbreviation section.

### DNA sequencing

Genomic DNA was isolated from muscle or fin using phenol-chloroform in the Autogen platform or DNeasy Tissue Extraction Kits (QIAGEN) following manufacturer’s instructions. The polymerase chain reaction (PCR) was used to obtain fragments of the mtDNA and nDNA and amplified using the primers compiled in Supplementary Data 4. PCRs for *COI*, *12s*, *16s*, *Atpase 8/6*, *361298E1*, *4174E20*, *55378E20*, and *S7-i1* were carried out for 10 µl volumes as follows: 1 µl of 10x buffer, 0.5 µl of 10 µM dNPTs, 0.4 µl of 50 µM MgCl_2_, 0.3 µl of 10uM of each primer, 5 U/M of Taq DNA polymerase, 6.4 µl of deionized water, and 1µl of DNA extract. Thermal cycling conditions for genes were: 35 cycles, 95 °C for 300 s, 95 °C for 30 s, 72 °C for 45 s, and 72 °C for 300 s. In the case of *ND4*, PCR was carried out for 20 µl volumes and 1 µl of DNA extract. Nested PCRs for *SH*_*3*_*PX*_*3*_ were carried out for 25 µl volumes and 2 µl of DNA extracts. Thermal cycling conditions for *SH*_*3*_*PX*_*3*_ were: 35 cycles, 94 °C for 60 s, 94 °C for 30 s, 72 °C for 80 s, and 72 °C for 300 s. The annealing temperatures and times are provided in Supplementary Data 4. PCR products were purified using EXOSAP. DNA sequencing followed standard protocols employed in molecular systematics laboratories and were completed through a capillary sequencing technique on the LAB MAHVN4550 sequencer. All obtained sequences were deposited in GenBank (Supplementary Data 3).

### Sequence alignment

Sequences were edited in the CodonCode Aligner (www.codoncode.com) and preliminarily aligned using ClustalW in MEGA 6.0.6.^40^. Alignments were checked by eye and manually adjusted when necessary. Kimura Two Parameter (K2P) pairwise distances were calculated using MEGA 6.0.6. For all analyses *Sternopygus*, when included, was designated as the outgroup. *Hypopomus artedi*, and four species of *Gymnotus* were also included. 107 individuals of *Electrophorus* from throughout their range were included as the ingroup. The best model of nucleotide evolution for each locus was estimated using jModelTest^41^, though codon-level estimates were not inferred or enforced. For introns sequences heterozygosity was noted with degenerate IUPAC codes. Insertion/deletion mutations for the EPIC *36298E1* sequences were incorporated in the phylogenetic analyses e.g., ref. ^42^.

### Species delimitation

Species delimitation was based on the subsequent evaluation of four molecular datasets. Dataset 1: Single locus (*COI*; 569 bp); Dataset 2: five mtDNA genes (*COI*, *ND4*, *ATP6/8*, *12S* rDNA, and *16S* rDNA; 2973 bp total); Dataset 3: five nDNA loci (one exon: *SH*_*3*_*PX*_*3*_; one intron: *S7-i1*; and three EPICs: *36298E20*, *4174E1*, 5*5378E20*; 2459 bp total); Dataset 4: concatenated mtDNA and nDNA genes (5432 bp).

Dataset 1: Application of a simple barcoding approach for species delimitation, i.e., *COI* sequences, in combination with pairwise distance comparisons has resulted in highly successful species-level identifications in fishes, e.g., ref. ^43^. In spite of this, determination of the limits between inter- and intra-specific differences and the delimitation of the appropriate level of differences between species threshold has proven difficult, particularly in under-sampled phylogenies^44^. For DNA taxonomy herein we utilize *COI*, which as noted above has previously demonstrated the ability to provide good resolution for species delimitation among fishes. We complement the traditional molecular taxonomic approach, i.e., a single tree for defining species clades and revealing the included gaps, with an independent investigative tool based on pairwise distances to automatically detect significant barcoding gaps without an a priori species hypothesis—the Automatic Barcoding Gap Discovery, ABGD^16^.

Dataset 2: A computationally multi-faceted parametric inferential approach. Species validation can come in many forms; herein our approach begins with a GMYC model using the 5-gene concatenated mtDNA. Given that branching events, in this case the history of haplotypes along any phylogenetic tree, should be more recent within a species and more distant between species; implementation of the GMYC model seeks to distinguish between cladogenetic (species-level differences modeled by the Yule process) and tokogenetic (intra-specific differences modeled by the Coalescent process) events. A Bayesian, single-threshold ML method^45^, and multi-threshold ML method^46^ are all available for the GMYC model and all three were used here. The single-threshold ML method is the most conservative approach, the multi-threshold method allows for variation in the depth of history at which tokogeny gives way to speciation^46^, and there is a Bayesian implementation that takes into account error in reconstruction of phylogeny and model uncertainty^47^. The full dataset (113 terminals) was reduced to unique haplotypes (63 terminals) prior to analyses. Beast 2.4^18^ was run for 20 million generations sampling every 1000 generations, and results were assessed using Tracer v1.5 (http://beast.bio.ed.ac.uk/Tracer) to ensure stationarity and to check that all parameters had acceptable effective samples sizes (>200) for use in generating the distribution of ultrametric trees. The derived maximum clade credibility (MCC) tree from the *BEAST2.4 run was used for the two GMYC analyses based on ML. These analyses were run on the GMYC web server (http://species.h-its.org/gmyc/) using the single and multi-threshold approaches, as described above. The Poisson tree process (PTP) has been shown to better delineate species, particularly when divergences among lineages is low^48^, and a Bayesian based implementation of this is available (http://species.h-its.org/ptp/). As two GMYC approaches were implemented we elected to use the bPTP method, to investigate a third approach to species number.

Dataset 3: Nuclear DNA used herein for *Electrophorus* and related species is a combination of three EPIC loci (see Supplementary Data 3), a single nuclear ribosomal intron (*S7-i1*), and one exon (*SH*_*3*_*PX*_*3*_). Further estimates of species boundaries and validation of species were completed by analyzing the data in several different ways including analyzing each nuclear locus individually to determine the posterior probability support provided by each locus, analyzing the concatenated full nuclear DNA Dataset 3, incorporating coalescent-based methods for species delimitation of nDNA loci via Bayesian Phylogenetics and Phylogeography (BP&P) v3.2^17^, and investigating the full ten locus dataset with the Genealogical Sorting Index, GSI^49^ using 10000 permutations on the lattice server^50^ as well as joint estimation of divergence times and the gene trees species tree using *BEAST2.4^18^. Details of these analyses are as follows: Each nuclear locus was analyzed individually and in a concatenated matrix using MrBayes v3.2.2^51^ on the CIPRES science gateway^52^ to determine the posterior probability for each of the putative lineages. The concatenated matrix was run for 10 million generations sampling every 1000 generations. Determination of convergence was completed with Tracer v1.5 and using Are We There Yet, AWTY (http://ceb.csit.fsu). Each individual nuclear and mitochondrial locus were run for 200 million generations sampling every 5000 generations using both MrBayes and *BEAST2.4. Stationarity of each locus in each run was assessed with Tracer and AWTY, and the distributions of the results from these runs were plotted in DensiTree v2.2.1^53^ with a ten percent burn-in to visualize the congruence and conflict among topologies across loci. Analyses were completed with the entire Dataset 4, but are visualized with only the focal taxon *Electrophorus*, and a reduced number of terminals in each putative species. This was completed by trimming the total number of terminals post-analysis using Phyutility^54^.

Dataset 4: Bayesian Phylogenetics and Phylogeography (BP&P) v3.2 was used to delimit species boundaries on the complete 10-locus dataset with all terminals included and with a reduced number of terminals (three individuals from each putative lineage). BP&P uses the multispecies coalescent to delimit species and infer phylogeny in a Bayesian framework. The program also accounts for population genetic uncertainties in incomplete lineage sorting associated with ancestral polymorphism conflicts in gene trees and species trees^55^. In this program the Gamma prior G (α,β) is assigned to both population size (θ) and age of the species tree root (τ_0_) and in our analyses α = 2 and β = 1000; all other parameters of divergence time used the Dirichlet prior^56^ with the heredity scalar set to 0.25 for the mtDNA loci and to 1 for the five nuclear loci. The analyses were run twice to ensure consistency between the runs.

### Phylogenetic estimation (trees with outgroups)

We estimated the phylogeny of *Electrophorus* using the concatenated alignment of dataset 4 (5852 nucleotides total) in RAxML and MrBayes 3.2.6^51^ both run on the CIPRES science gateway^52^.

Time divergence estimates: We simultaneously estimated divergence time, based on an external calibration point, and the species trees from multilocus sequence data using *BEAST2.4 and a relaxed clock for the mtDNA loci and a strict clock for the nDNA^23^. A subject of much recent debate has been the chronological closure of the Central American Seaway via the Isthmus of Panama and its consequences for biotic dispersal between North and South America and vicariance between Atlantic and Pacific Oceans^57–64^. On the younger side, ^62^dated the formation of the Isthmus of Panama *sensu stricto* to around 2.8 Ma. Paleoceanographic studies^20,65^ show a decrease in the transport of deep and intermediate Pacific waters into the Caribbean by 10 to 11 Ma, probably related to a closing Central American Seaway^61^. Based on uranium-lead geochronology in detrital zircons, ^61^provided evidence that rivers originating on the Panama arc transported sediment to the shallow marine basins of northern South America by the middle Miocene (13–15 Ma). Finally, ^57^used both molecular and fossil data to argue for two significant waves of terrestrial dispersal at around 20 and 6 Ma. Based on these studies there a wide time scale from which to select calibration points, each with support from the literature: 2.8 Ma^62^; and 5.1 Ma—^57,58^final wave of colonization; 10.5 Ma—^20,65^closing of Central American Seaway; 14 Ma – ^61^fluvial transport of sediment from Panama arc to South America; or 20 Ma—^57,58^early wave of terrestrial dispersal. The date used, i.e., 10.5 ± 1.5 Ma, is in the middle of these ranges and is a conservative approach as well as one supported by multiple studies. Mitochondrial DNA indels. One other item of note with respect to the molecular sequence data generated for this study concerns the mitochondrial gene *ND4* and indels. In the aligned data matrix *Hypopomus artedi* was found to have a full codon triplet in *ND4* that no other sampled member of the outgroup or ingroup contained. Full codon gaps are known in the mtDNA of fishes, e.g., *ND2* of *Aphredoderus*^66^. It is unlikely that this amplicon is a pseudogene as no stop codons in either this sequence or any outgroup or ingroup sequences were demonstrated by ORFfinder at NCBI. In addition, at position 1270 (−569) within the species of *Electrophorus* some individuals have an extra adenine residue.

### Molecular diagnosis

Species of *Electrophorus* were diagnosed by unique nucleotide substitutions shared by all individuals of the distinct populations. Optimizations of the nucleotide substitutions among the species of *Electrophorus* were obtained from the MP topology using MEGA 6.0.6. Each numeric position was determined by the alignment between the species of *Electrophorus* with the outgroup. Screening for diagnosed nucleotide substitution were performed manually post alignment using Mesquite (http://mesquiteproject.org).

### Phenotypic analysis

Morphometric and meristic summaries do not include data from individuals smaller than 300 mm TL. Although of large to very large sizes compared to most species of Neotropical freshwater fishes, specimens of *Electrophorus* less than 300 mm are juveniles with pronounced differences in some meristic (e.g., number of anal/caudal-fin rays) and morphometric values (e.g., preanal-fin distance) relative to larger specimens. Internal anatomy was studied through radiographs.

Meristics follow^11^ with the addition of the number of lateral-line pores posterior of the gill opening. Anal/caudal-fin ray counts include the dorsal procurrent rays, when present (made through radiographs). Morphometrics are point-to-point distances taken with digital calipers with intra-specific ranges presented in tables. Measurements were taken from the left side of individuals, when possible, as follows: body width—the distance across the body at the pectoral-fins base; branchial opening—the distance from the dorsal to the ventral extremities of the opening; eye diameter—the horizontal distance between the anterior and the posterior margins of the eye; eye-posterior naris distance—the distance from the anterior margin of the eye to the posterior margin of the posterior naris; greatest body depth—the greatest vertical extent of the body, usually at the origin of the anal fin along the posterior margin of the gill slit; head depth—the distance between the dorsal and ventral margins of the head at the vertical through the eye; head length—the distance from the tip of the lower jaw to the posterior margin of the opercle; head width—the horizontal distance between the dorsal limits of the branchial opening; internarial distance—the distance between the posterior margin of the anterior naris and the anterior margin of the posterior naris; interorbital distance—the distance between the medial margins of the eyes; mouth-eye distance—the distance from the posterior margin of the mouth to the ventral margin of the eye; mouth width—the distance between the inner corners of the mouth; pectoral-fin length—the distance from the base of the dorsal most pectoral-fin ray to the distal most point on the fin margin; postorbital distance—the distance from the posterior margin of the eye to the posterior margin of the opercle; preanal-fin distance—the distance from the tip of the lower jaw to the anal-fin origin; preanus distance—the distance from the tip of lower jaw to the anterior margin of the anus; preorbital distance—the distance from the anterior margin of the eye to the anterior margin of the lower jaw; snout-corner of mouth distance—the distance from the snout to the corner of the mouth; and total length—the distance from the tip of the lower jaw to the base of the central caudal-fin ray.

### Species distribution modelling and niche analysis

According to^67^ the species distribution patterns are the consequences of three main factors: (1) dispersal ability; (2) the spatial distribution of environmental conditions that determine the survival of individuals and the persistence of populations; and (3) biotic interactions and the dynamics of resources. The species distribution models are based on the set of climate variables in wide resolution scales (macroscale) that determine the distribution of organisms, i.e., Grinnelian niche^67^.

To build the SDM models we used the MaxEnt algorithm, which works with presence data only^68^. Methods that use only presence data are common especially in areas with large gaps of information and high biodiversity such as the Amazon River basin, where there is no information about absence. MaxEnt estimates the probability of species distribution by fitting a function close to the uniform distribution under the environmental information associated to the occurrence points^68^. This method can discriminate between the environmental variables associated to the occurrence data and the background variation of the predictor variables based on 10000 random points, i.e., the algorithm contrasts presences against the background location^69^. We used occurrence points of the three species described in this paper, to show the niche differences among them, *Electrophorus electricus* had 29, *E*. *voltai* 24, and *E*. *varii* 46 spatially unique occurrence points, i.e., one occurrence point per pixel, split into 20% test and 80% training.

The environmental variables were chosen according to their potential to represent the topographical and limnological characteristics in the Amazon freshwater ecosystem based on^70^ who showed that broad scale variables could be used as proxies for characteristics of the local aquatic environment for modelling fish species distributions in areas like the Amazon where large gaps exist in our understanding of distributions. The climatic macroscale variables were obtained from BioClim (www.worldclim.org): annual mean precipitation (AMP), annual mean temperature (AMT), seasonality of precipitation (SP) and seasonality of temperature (ST). We also used geomorphological variables about terrain slope (SL), altitude (AL) and flow accumulation (FLA) obtained from Hydro1k (www.usgs.gov) database. Soil type characteristics (SOT 0, 3, 6, 11) were gathered from FAO’s database (www.fao.org.br). All descriptor variables were obtained for pixels of 4 × 4 km of resolution.

Model evaluation was performed using the Area Under Curve (AUC), which is a threshold-independent measure based on ranking locations, i.e. the probability to choose randomly the presence locations in relation to randomly choosing the background locations, commonly used in SDM modelling^71,72^. This measure could be interpreted as average of true positives values (sensitivity) of all possible false positive values (specificity), producing a global measure of fit for the model. These values are then plotted (sensitivity against 1-specificity) to generate what is known as the ROC (Receiver Operating Characteristic) curve^72^. Alternatives to AUC there are the threshold dependent measures that from a threshold create a presence and absence binary feature and a confusion matrix^71^. For that we used the Minimum Difference Threshold Criterion (MDT) that are expected to minimize omission and commission errors^73^.

To test the significance of the niche differentiation among lineages, we performed the multivariate analyses of variance (MANOVA). All the analyses were performed using dismo and vegan packages for Maxent, and MANOVA in R software^74^.

### Electric organ discharge analysis

Low-voltage EOD waveform recordings. We measured the low-voltage electrolocation pulses generated irregularly (rates of ca. 0.1–10 Hz) by the Sachs’ electric organ^5^. Head-to-tail EOD waveforms sensu ref. ^75^ were recorded within 12 h of capture in inflatable swimming pools (2.5–3.0 m diameter, or rectangular ca. 3 × 1.8 m) filled to 40 cm depth with water from the collecting site. Temperature was standardized to 27.0 ± 0.2 °C. Submerged NiCr electrodes were placed at least 40 cm away from the head and tail of the fish, along the head–tail axis, and a train of low-voltage pulses acquired directly by an audio-digitizer (96 kHz sampling rate) or a National Instruments digital acquisition device (sampling rates 100–200 kHz). Here we plot a single low-voltage EOD for each individual. We measured EOD duration at a 1% threshold of the peak positive amplitude of the EOD following ref. ^75^.

High-voltage EOD amplitudes. We measured the high-voltage pulses generated in rapid volleys by the main electric organ and Hunter’s electric organ for predation and defense^4^. We used a Fluke 190–202 storage oscilloscope to measure the peak voltage in the volley of high-voltage EODs. Soon after capture the subject specimen was stretched out on a dry heavy-duty (non-conductive) plastic sheet to isolate it from the load of water. In this position a DC-coupled voltage reading from snout to the distal end of the tail was taken by gently prodding the tip of the snout to elicit a volley of high-voltage discharges. The entire procedure was accomplished in less than one minute.

Low-voltage EOD quantitative analysis. All seven EODs were conditioned to a common sampling rate, energy-normalized to root mean squared (rms) amplitude and centered to the peak of the single EOD phase. Following the procedure described in refs. ^30,32^, and using a custom MATLAB (The Mathworks, Natick, MA) program, we subjected the conditioned waveforms to the discrete wavelet transform (DWT), using the Symmlet-4 wavelet base, to generate a matrix of 256 DWT coefficients (256 unique coefficients at 8 wavelet scales [(2^8) −1], and one scaling coefficient). The DWT is a popular time-frequency based procedure to deconstruct signals into a smaller number of features informative of temporal (waveform shape) and spectral (frequency) differences among groups of signals^76^. Following ref. ^30^ we then subjected the matrix of 256 DWT coefficients × 7 individuals to dimension reduction by pairwise ANOVA to extract those waveform features (DWT coefficients), which permit the most effective discrimination among the three species. This yielded a final matrix of just 4 DWT coefficients × 7 individuals. Finally, using the ‘cluster’ package in Statistica 13.3 (Tibco/Statsoft, Palo Alto, CA) we performed nearest-neighbor (single linkage) hierarchical clustering of all individual EODs in the matrix of reduced DWT coefficients, with the Euclidean distance as a metric of distance in multivariate space.

### Nomenclatural acts

This published work and the nomenclatural acts it contains have been registered in ZooBank, the proposed online registration system for the International Code of Zoological Nomenclature (ICZN). The ZooBank LSIDs (Life Science Identifiers) can be resolved and the associated information viewed through any standard web browser by appending the LSID to the prefix “http://zoobank.org/”. The LSIDs for this publication are: 7598B3E4-8E0C-43CE-A57B-CD71A9C99526, 7FA17DC2-5F58-4366-8908-9E66BE922458, and 142863F0-1F6F-4789-A05B-ECECC3CC022F.

### Reporting summary

Further information on research design is available in the Nature Research Reporting Summary linked to this article.

## Supplementary information

Supplementary Information

Reporting Summary

Description of Additional Supplementary Files

Supplementary Data 1

Supplementary Data 2

Supplementary Data 3

Supplementary Data 4

## Data Availability

Sequences for all molecular markers are available from the GenBank database (accession numbers are listed in Supplementary Data 3). Specimens from which DNA samples were analyzed were deposited along with tissue samples at the biodiversity collections listed in Supplementary Data 1. All data are available upon reasonable request.
