# Supplementary material for: Unexpected species diversity in electric eels with a description of the strongest living bioelectricity generator
Source: Nat Commun. 2019 Sep 10;10:4000. doi: 10.1038/s41467-019-11690-z (PMC6736962; doi:10.1038/s41467-019-11690-z)
Supplement: Supplementary file 1 — Supplementary Information [file 41467_2019_11690_MOESM1_ESM.pdf]

**Supplementary Information**

# Unexpected species diversity in electric eels with a description of the strongest living bioelectricity generator

de Santana et al.

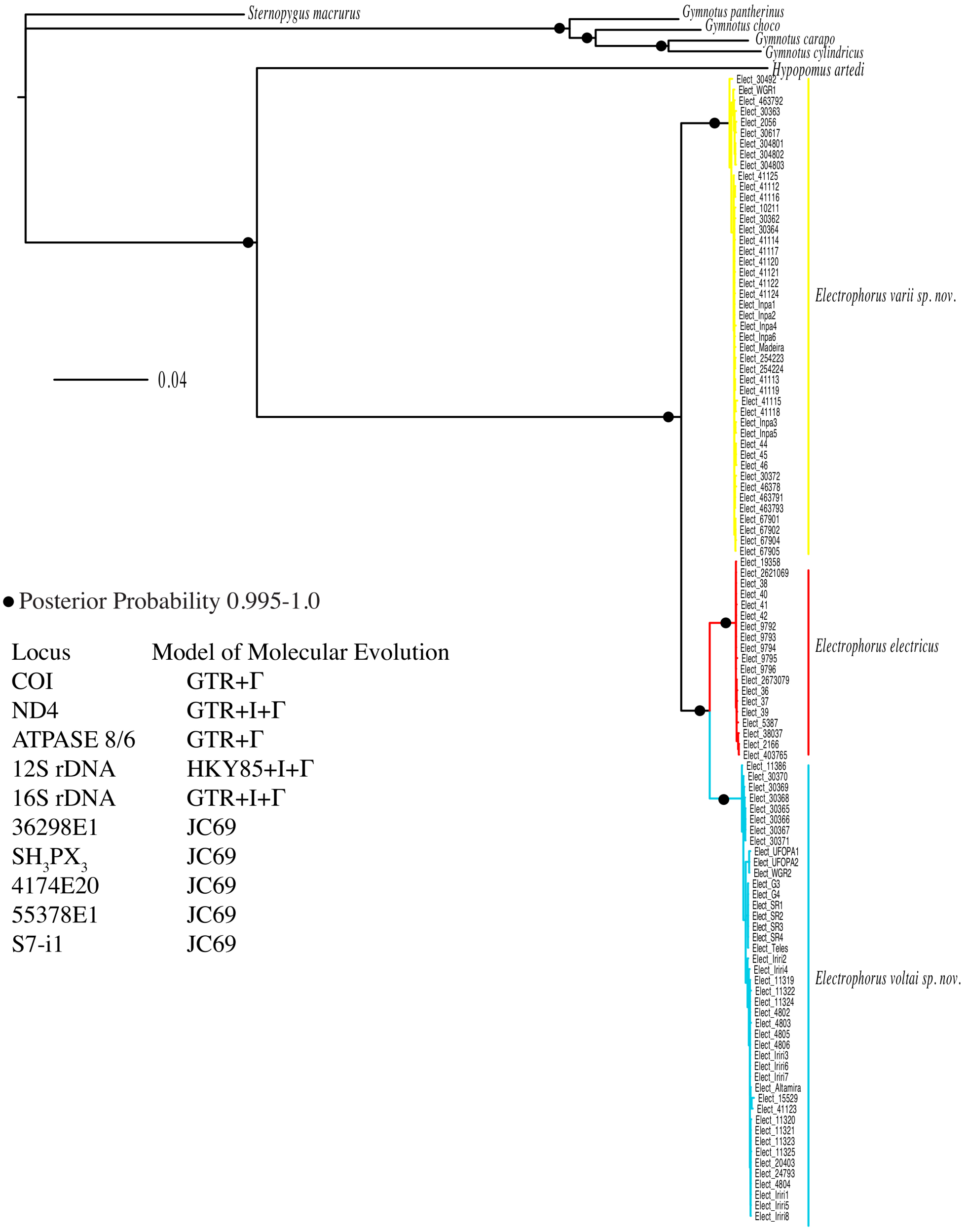


● Posterior Probability 0.995-1.0

| Locus | Model of Molecular Evolution |
|---|---|
| COI | GTR+Γ |
| ND4 | GTR+I+Γ |
| ATPASE 8/6 | GTR+Γ |
| 12S rDNA | HKY85+I+Γ |
| 16S rDNA | GTR+I+Γ |
| 36298E1 | JC69 |
| $SH_3PX_3$ | JC69 |
| 4174E20 | JC69 |
| 55378E1 | JC69 |
| S7-i1 | JC69 |

**Supplementary Figure 1.** Tree of life for individuals of *Electrophorus* and outgroups**.** The majority-rule consensus topology for a Bayesian inferred phylogenetic hypothesis of *Electrophorus* based on the concatenated ten locus data set using MrBayes v3.2.6. Species of *Electrophorus* are color-coded by species inferred. All labelled nodes are supported by more than 0.995. Many nodes inside of each species have strong posterior probability support but branch lengths when compared to the outgroup are very short and therefore difficult to display. Model of molecular evolution is given for each locus.
