# Supplementary material for: Unexpected species diversity in electric eels with a description of the strongest living bioelectricity generator
Source: Nat Commun. 2019 Sep 10;10:4000. doi: 10.1038/s41467-019-11690-z (PMC6736962; doi:10.1038/s41467-019-11690-z)
Supplement: Supplementary file 2 — Reporting Summary [file 41467_2019_11690_MOESM2_ESM.pdf]

nature research

Corresponding author(s): Carlos David de Santana

Last updated by author(s): Jul 25 2019

Reporting Summary

Nature Research wishes to improve the reproducibility of the work that we publish. This form provides structure for consistency and transparency in reporting. For further information on Nature Research policies, see Authors & Referees and the Editorial Policy Checklist.

Statistics

For all statistical analyses, confirm that the following items are present in the figure legend, table legend, main text, or Methods section.

n/a	Confirmed	
☐	☒	The exact sample size (*n*) for each experimental group/condition, given as a discrete number and unit of measurement
☐	☒	A statement on whether measurements were taken from distinct samples or whether the same sample was measured repeatedly
☒	☐	The statistical test(s) used AND whether they are one- or two-sided<br>*Only common tests should be described solely by name; describe more complex techniques in the Methods section.*
☐	☒	A description of all covariates tested
☒	☐	A description of any assumptions or corrections, such as tests of normality and adjustment for multiple comparisons
☒	☐	A full description of the statistical parameters including central tendency (e.g. means) or other basic estimates (e.g. regression coefficient) AND variation (e.g. standard deviation) or associated estimates of uncertainty (e.g. confidence intervals)
☒	☐	For null hypothesis testing, the test statistic (e.g. *F*, *t*, *r*) with confidence intervals, effect sizes, degrees of freedom and *P* value noted<br>*Give P values as exact values whenever suitable.*
☐	☒	For Bayesian analysis, information on the choice of priors and Markov chain Monte Carlo settings
☐	☒	For hierarchical and complex designs, identification of the appropriate level for tests and full reporting of outcomes
☒	☐	Estimates of effect sizes (e.g. Cohen's *d*, Pearson's *r*), indicating how they were calculated

Our web collection on statistics for biologists contains articles on many of the points above.

Software and code

Policy information about availability of computer code

Data collection	No software was used.
Data analysis	CodonCode Aligner; Mesquite v3.0.3; jModelTest; ArcGIS; MEGA v6.0.6; Automatic Barcode Gap Discovery; GMYC web Server; BP&P v3.2 ; RAxML; MrBayes v3.2.6; *BEAST2.4; Tracer v1.5; AWTY; DensiTree v2.2.1; Phyutility; R Development Core Team; MATLAB; Statistica v13.3.

For manuscripts utilizing custom algorithms or software that are central to the research but not yet described in published literature, software must be made available to editors/reviewers. We strongly encourage code deposition in a community repository (e.g. GitHub). See the Nature Research guidelines for submitting code & software for further information.

Data

Policy information about availability of data

All manuscripts must include a data availability statement. This statement should provide the following information, where applicable:

- Accession codes, unique identifiers, or web links for publicly available datasets
- A list of figures that have associated raw data
- A description of any restrictions on data availability

Sequences for all molecular markers are available from the GenBank database (accession numbers are listed in Supplementary Data 3). Specimens from which DNA samples were analyzed were deposited along with tissue samples at the biodiversity collections listed in Supplementary Data 1. All data are available upon reasonable request.

Field-specific reporting

Please select the one below that is the best fit for your research. If you are not sure, read the appropriate sections before making your selection.

☐ Life sciences ☐ Behavioural & social sciences ☒ Ecological, evolutionary & environmental sciences

For a reference copy of the document with all sections, see nature.com/documents/nr-reporting-summary-flat.pdf

Ecological, evolutionary & environmental sciences study design

All studies must disclose on these points even when the disclosure is negative.

Study description	Our study tested the hypothesis of a single species of electric eel in the genus Electrophorus. Based on patterns of genetic, morphological, and ecological data, we rejected the hypothesis of a single species broadly distributed throughout Greater Amazonia. Our analyses promptly identified three major lineages species of electric eel. One of new species is the strongest living bioelectricity generator.
Research sample	Specimens of widespread populations of Electrohorus electricus, including those from the type locality in Suriname.
Sampling strategy	We aimed to have at last one specimen per locality across the distributional range of Electrophorus electricus.
Data collection	Specimens and tissues samples were collected by Carlos David de Santana, William GR Crampton, Mark H Sabaj, Raphaël Covain, Jonathan Ready, Jansen Zuanon, Renildo R. de Oliveira, Raimundo N. Mendes-Júnior, Douglas A. Bastos, Tulio F. Teixeira, Jan Mol, Willian Ohara, Luiz A. Peixoto, Cleusa Nagamachi, Leandro Sousa, Luciano F. A. Montag, Frank Ribeiro, and Nivaldo M. Piorsky.
Timing and spatial scale	Specimens were collected in localities (See Supplementary Data 1) across South America during the dry season. Most of samples were collected from 2014 to 2017.
Data exclusions	Morphometric and meristic summaries do not include data from individuals smaller than 300 mm TL. Although of large to very large sizes compared to most species of Neotropical freshwater fishes, specimens of Electrophorus less than 300 mm are juveniles with pronounced differences in some meristic (e.g., number of anal/caudal-fin rays) and morphometric values (e.g., preanal-fin distance) relative to larger specimens. Some adult specimens were damaged and morphometric and meristics accounts were not taken. A small number of DNA samples could not be amplified for specific genes (See Supplementary Data 3).
Reproducibility	We report all protocols for the generation of data in Methods and have deposited data in the Genbank. Upon request, we can provide aligned sequences.
Randomization	We performed evolutionary analyses that did not allow for randomization
Blinding	Not applicable. We performed an evolutionary analyses and so blinding was not relevant

Did the study involve field work? ☒ Yes ☐ No

Field work, collection and transport

Field conditions	Specimens and tissue samples were collected during fieldwork in the Amazon rainforest.
Location	Fishes were collected in rivers in the Greater Amazonia, South America. See Supplementary Data 1.
Access and import/export	Material was collected following countries and international guidelines and with appropriated permits.
Disturbance	No disturbance was caused.

Reporting for specific materials, systems and methods

We require information from authors about some types of materials, experimental systems and methods used in many studies. Here, indicate whether each material, system or method listed is relevant to your study. If you are not sure if a list item applies to your research, read the appropriate section before selecting a response.

Materials & experimental systems

n/a	Involved in the study
☒	☐ Antibodies
☒	☐ Eukaryotic cell lines
☒	☐ Palaeontology
☐	☒ Animals and other organisms
☒	☐ Human research participants
☒	☐ Clinical data

Methods

n/a	Involved in the study
☒	☐ ChIP-seq
☒	☐ Flow cytometry
☒	☐ MRI-based neuroimaging

Animals and other organisms

Policy information about studies involving animals; ARRIVE guidelines recommended for reporting animal research

Laboratory animals	The study did not involve laboratory animals.
Wild animals	Specimens of Electrophorus were caught using hand nets, seine nets, and fishing spears. Individuals were sacrificed using MS 222. Tissue samples were collected and preserved in alcohol. Specimens were fixed in 10% formalin and preserved in 70% alcohol. Specimens and tissues were deposited in natural history collections around the world.
Field-collected samples	Soon after capture the subject specimen was stretched out on a dry heavy-duty (non-conductive) plastic sheet to isolate it from the load of water. In this position a DC-coupled voltage reading from snout to the distal end of the tail was taken by gently prodding the tip of the snout to elicit a volley of high voltage discharges. The entire procedure was accomplished in less than one minute.
Ethics oversight	Specimens were collected and sampled in the field according to the Animal Care and Use standards of the depository institutions and the countries of origin of the tissue samples used in the DNA analyses. In addition, tissues and/or specimens were received from multiple institutions in North and South America and Europe following pertinent Material Transfer Agreements and the national and international protocols for the shipment of materials.

Note that full information on the approval of the study protocol must also be provided in the manuscript.
