# Supplementary material for: Unexpected species diversity in electric eels with a description of the strongest living bioelectricity generator
Source: Nat Commun. 2019 Sep 10;10:4000. doi: 10.1038/s41467-019-11690-z (PMC6736962; doi:10.1038/s41467-019-11690-z)
Supplement: Supplementary file 3 — Description of Additional Supplementary Files [file 41467_2019_11690_MOESM3_ESM.pdf]

Description of Additional Supplementary Files

File Name: Supplementary Data 1
Description: Localities for 107 specimens of *Electrophorus*.

File Name: Supplementary Data 2
Description: Morphometric and meristic data for specimens of *Electrophorus electricus*, and holotypes and paratypes of *Electrophorus varii* sp. nov. and *Electrophorus voltai* sp. nov. N = number of individuals. H = holotype.

File Name: Supplementary Data 3
Description: List of sequenced genes, Genbank accession numbers for 107 specimens of *Electrophorus* and six outgroup species.

File Name: Supplementary Data 4
Description: List of primers, annealing temperature and time, ingroup and outgroup obtained fragment sizes, and sources.
